# Exclusive transmission of the embryonic stem cell‐derived genome through the mouse germline

**DOI:** 10.1002/dvg.22938

**Published:** 2016-05-18

**Authors:** Frank Koentgen, Jiangwei Lin, Markella Katidou, Isabelle Chang, Mona Khan, Jacqui Watts, Peter Mombaerts

**Affiliations:** ^1^ Ozgene Pty LtdBentleyWestern Australia6983Australia; ^2^ Max Planck Research Unit for NeurogeneticsMax‐von‐Laue‐Strasse 4Frankfurt60438Germany

**Keywords:** embryonic stem cell, sterility, Tsc22d3, Gilz

## Abstract

Gene targeting in embryonic stem (ES) cells remains best practice for introducing complex mutations into the mouse germline. One aspect in this multistep process that has not been streamlined with regard to the logistics and ethics of mouse breeding is the efficiency of germline transmission: the transmission of the ES cell‐derived genome through the germline of chimeras to their offspring. A method whereby male chimeras transmit exclusively the genome of the injected ES cells to their offspring has been developed. The new technology, referred to as goGermline, entails injecting ES cells into blastocysts produced by superovulated homozygous *Tsc22d3* floxed females mated with homozygous *ROSA26‐Cre* males. This cross produces males that are sterile due to a complete cell‐autonomous defect in spermatogenesis. The resulting male chimeras can be sterile but when fertile, they transmit the ES cell‐derived genome to 100% of their offspring. The method was validated extensively and in two laboratories for gene‐targeted ES clones that were derived from the commonly used parental ES cell lines Bruce4, E14, and JM8A3. The complete elimination of the collateral birth of undesired, non‐ES cell‐derived offspring in goGermline technology fulfills the reduction imperative of the 3R principle of humane experimental technique with animals. genesis 54:326–333, 2016. © 2016 The Authors. Genesis Published by Wiley Periodicals, Inc.

## INTRODUCTION

Gene targeting in mice via homologous recombination in embryonic stem (ES) cells has been extraordinarily informative in all fields of biomedical research (Capecchi, 2008; Evans, 2008; Smithies, 2008). The novel technology of CRISPR‐Cas9 can introduce gene edits directly into the genome of the mouse zygote and thereby obviates the intermediary vehicle of ES cells (Yang *et al*., 2014). But for complex genetic modifications such as floxed alleles, bicistronic mutations, and knockins of large DNA segments, gene targeting in ES cells, as we know it since the late 1980s, remains best practice.

One aspect in this long multistep process that has not been streamlined with regard to the logistics and ethics of mouse breeding is the efficiency or rate of germline transmission (GLT): the transmission of the ES cell‐derived genome through the germline of male chimeras to their offspring (Bradley *et al*., 1984). Such chimeras are referred to as germline chimeras. It is not possible to screen for germline transmission other than by the biological test of breeding the chimeras, but numerous undesired, non‐ES cell‐derived offspring are hereby born collaterally. Male chimeras are typically selected for breeding on the basis of high somatic ES cell contribution as assessed visually by coat color chimerism, and germline offspring are identified by coat color. Among the ES cell‐derived offspring, 50% inherit the targeted mutation, if it is present in a heterozygous state in the injected ES cells. But it remains difficult to predict the rate of GLT for an individual chimera or for a set of chimeras generated with a particular gene‐targeted ES cell clone. Overbreeding may be the result, in particular when there is urgency in establishing a novel gene‐targeted strain: too many chimeras are set up for breeding, too many offspring are sired, too many non‐ES cell derived offspring are born and these are of no further use and are typically culled. A substantial fraction of injected ES cell clones result in chimeras that sire only undesired, non‐ES cell‐derived offspring.

Over the years attempts have been made to improve the rate of GLT, but none of these methods have become standard practice. Chimeras with 100% GLT were already reported in 1993 by aggregating ES cells with tetraploid blastocysts (Nagy *et al*., 1993) but the birth rate is low. The Perfect Host approach (Taft *et al*., 2013) promised to improve GLT rates by generating male chimeras with diphtheria‐toxin mediated ablation of the host germline. But this approach, as it was described, is imperfect in that it disregards the practical advantages of coat color differences between strains used to derive the ES cell lines and strains used to produce blastocysts. Moreover the Perfect Host approach was tested out only for 11 gene‐targeted ES cell clones and in only one laboratory. There is no report in the literature that has made since then use of this approach.

Here we report the development of a new technology, called goGermline, that affords 100% GLT by male chimeras. The technology is based on the unexpected observation that males with a mutation in the gene *Tsc22d3*, also called *Gilz*, are completely sterile due to a cell‐autonomous defect in spermatogenesis, and relies further on the convenient location of *Tsc22d3* on the X chromosome. When colonizing the germline of hemizygous *Tsc22d3* knockout males, cells that descended from the injected ES cells have no competition from cells that descended from the host embryo. The male chimeras can be sterile but, when fertile, they transmit the ES cell‐derived genome to 100% of their offspring. Fertile chimeras thus behave genetically as heterozygotes. The technology is so efficient that in our current standard operating protocol, a project is considered successful as soon as females mated with chimeras are observed to be pregnant; no further injections need to be scheduled, and these or additional chimeras need not be bred further.

In conclusion, goGermline technology eliminates entirely the collateral birth of undesired, non‐ES cell‐derived offspring.

## RESULTS

### The *Tsc22d3* Gene

The X‐linked gene *Tsc22d3*, also called *Gilz* (D'Adamio *et al*., 1997), encodes a leucine zipper protein that unexpectedly was found to be essential for male fertility. Males hemizygous for a knockout mutation in *Tsc22d3*, from three independently generated strains (Bruscoli *et al*., 2012; Ngo *et al*., 2013a, 2013b; Romero *et al*., 2012; Suarez *et al*., 2012), are sterile due to the inability of spermatocytes to complete the first meiotic division. A few weeks after birth, hemizygous *Tsc22d3* knockout males display Sertoli‐cell‐only seminiferous tubuli, which are totally devoid of germ cells. Importantly, wild‐type germ cells transplanted into the testes of hemizygous *Tsc22d3* knockout males can repopulate seminiferous tubuli (Bruscoli *et al*., 2012), suggesting that these testes can still support normal spermatogenesis. We thus reasoned that the testicular environment of hemizygous *Tsc22d3* knockout males should be conducive to germ cell differentiation of cells that descended from the injected ES cells.

**Figure 1:**
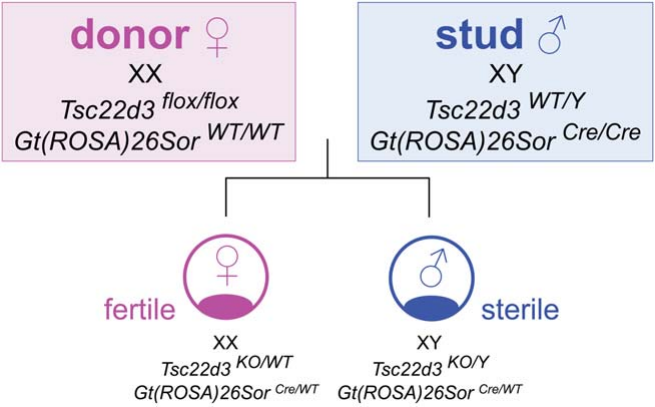
Schematic of goGermline technology. Blastocysts are produced by mating a superovulated homozygous *Tsc22d3* floxed female (donor) with a homozygous *ROSA26‐Cre* male (stud). Male blastocysts develop into sterile males, but fertility can be rescued by injecting ES cells. The resulting chimeras, if fertile, produce exclusively ES cell‐derived offspring (100% GLT). Female blastocysts produce fertile females, and these are not bred.

It is obviously not possible to maintain a homogeneous knockout strain if the males of this strain are sterile. Moreover, to streamline the logistics of strain maintenance, we wanted to develop a method whereby no genotyping of the females and males used for blastocyst production is ever necessary. We solved this double challenge by applying a conditional knockout strategy: we cross females homozygous for a floxed *Tsc22d3* mutation with males homozygous for a targeted *ROSA26‐Cre* mutation (Fig. 1). Both strains are homozygous viable, healthy, and fertile. Male offspring of this cross carry the *Tsc22d3* mutation on their X chromosome. The *loxP*‐flanked segment gets excised in all cells including the germline by the enzymatic action of the Cre recombinase, which is expressed ubiquitously from the *ROSA26* locus. These males are sterile, and can also replace vasectomized males to condition pseudopregnant recipient females.

### goGermline in Setting #1

We tested goGermline technology for two commonly used parental ES cell lines, E14 (Handyside *et al*., 1989) and JM8A3.N1 (Pettitt *et al*., 2009), in the setting of an academic laboratory (Max Planck Research Unit for Neurogenetics). The blastocysts were generated by mating superovulated homozygous *Tsc22d3* floxed, BALB/c × albino‐agouti C57BL/6 F1 or F2 females with homozygous *ROSA26‐Cre* BALB/c males. (The various strain configurations are described below and in the “Methods” section.) We illustrate the application of goGermline technology in this setting with two projects by way of examples.

**Figure 2:**
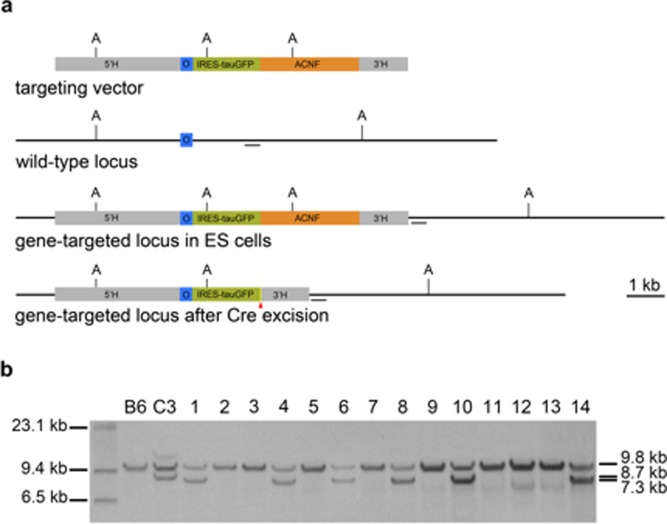
The OMP‐IRES‐tauGFP strain. (**a**) Construction of a gene‐targeted strain with an OMP‐IRES‐tauGFP mutation. 5′H, 5′ homology arm; O, the intronless OMP coding sequence; ACNF, neomycin selectable marker; 3′H, 3′ homology arm; A, *ApaL*I restriction site. The *short horizontal bar* denotes the external probe for Southern blot hybridization. The *red triangle* represents the *loxP* site that remains in the locus after self‐excision of the *ACNF* cassette during passage through the male germline. The final result is a bicistronic mutation whereby cells that express OMP also express tauGFP. (**b**) Non‐radioactive Southern blot hybridization of genomic DNA from the first litter (samples 1–14) produced by a goGermline chimera of clone C3. Six out of 14 mice are heterozygous. Genomic DNA prepared from the liver was digested with *ApaL*I. The fragment representing the wild‐type allele is 9.8 kb, the fragment representing the mutant allele in ES clone C3 is 8.7 kb, and the fragment representing the mutant allele in the heterozygous mouse after self‐excision of the *ACNF* cassette during passage through the male germline, is 7.3 kb.

In a first project, we injected home‐made ES cell clones carrying a targeted bicistronic mutation in the *Omp* gene, which is expressed selectively in mature olfactory sensory neurons (Potter *et al*., 2001). The internal ribosome entry site enables cotranslation of OMP with the axonal marker tauGFP (Fig. 2a). Cells of targeted clones C3, C8, and C47 from parental ES cell line E14 (background 129P2/OlaHsd, chinchilla coat color, *Tyr^c‐ch^/Tyr^c‐ch^* and *A^w^/A^w^*) were injected into, respectively, 22, 34, and 22 blastocysts. We obtained, respectively, 12, 18, and 10 pups (total 40 pups out of 78 transferred blastocysts, or 51%), of which, respectively, 11, 12, and 3 were chimeric (total 26 chimeras out of 40 pups, or 65%). Chimerism could be assessed easily from the coat color differences on the albino background. There were, respectively, 4, 7, and 0 male chimeras, and 7, 5, and 3 female chimeras. Three male chimeras from clone C3 were set up for breeding with C57BL/6J females, and two sired 61 offspring (all agouti), of which 26 are heterozygous for the targeted mutation (Fig. 2b). Three male chimeras from clone C8 were set up for breeding with C57BL/6J females, and one sired 13 offspring (all agouti), of which five carried the targeted mutation. The rate of heterozygosity among offspring is consistent with 100% GLT: the goodness of fit (Chi‐square test) gives a *P*‐value of 0.16 for 31 heterozygotes among 74 offspring.

A second project concerned KOMP clone B07 from parental ES cell line JM8A3.N1 (background C57BL/6N, +/+, and A/a), which carries a knockout mutation in the *Meis2* gene. Using the conventional method of chimera generation by injecting B07 cells into C57BL/6J blastocysts, we had previously obtained only one male chimera with high coat color chimerism. This chimera sired 186 agouti or black offspring when mated with C57BL/6J females. (The JM8A3.N1 parental ES cell line, which is widely used in the KOMP project, has the non‐agouti mutation repaired but on only one chromosome, resulting in germline offspring that are agouti or black when male chimeras are crossed with C57BL/6J or C57BL/6N females. All offspring must thus be genotyped.) Only 7 of these 186 offspring were found to be heterozygous, and only 3 of these 7, all females, survived to adulthood. We, thus, surmised that heterozygosity for the *Meis2* mutation causes perinatal lethality. We proceeded to inject B07 cells into 64 goGermline blastocysts. The 19 pups born were all chimeric. Of the 14 male chimeras, 8 were set up for breeding with C57BL/6J females, and 4 sired 77 agouti or black offspring, among which only 3 (one agouti male, one black male, and one black female) were heterozygous; the agouti male died at 2 months. Having confirmed our suspicion of perinatal lethality of *Meis2* heterozygous mice, we sacrificed three C57BL/6J females late in gestation, and genotyped 16 out of 25 embryos as heterozygous. This rate of heterozygosity is consistent with 100% GLT: the goodness of fit (Chi‐square test) gives a *P*‐value of 0.16 for 16 heterozygotes among 25 embryos. We, thus, capitalized on the exclusive germline transmission of the ES cell‐derived genome in goGermline male chimeras: these mice behave effectively as heterozygotes.

### goGermline in Setting #2

In order to take full advantage of coat color differences for identifying chimeras and ES cell‐derived offspring, and to benefit from high and consistent yields of blastocysts by superovulating hybrid instead of inbred mice, we constructed several specialized strains (see “Methods” section for details.)

We tested three configurations of female blastocyst donors homozygous for the floxed *Tsc22d3* mutation. The first configuration is an F1 or F2 of BALB/c × albino‐agouti C57BL/6 (*Tyr^c^*/*Tyr^c^* and A/A at the albino and agouti loci); the second configuration is C57BL/6 (+/+ and a/a); and the third configuration is an F1 of BALB/c × C57BL/6 (*Tyr^c^*/+ and A/a). In all three configurations, studs were homozygous for the *ROSA26‐Cre* mutation and BALB/c (*Tyr^c^*/*Tyr^c^* and A/A), and chimeras were bred with C57BL/6 females (+/+ and a/a).

Table 1 summarizes the data obtained at Ozgene with 216 gene‐targeted clones from parental ES cell line Bruce4 (background C57BL/6‐Thy1.1, +/+, and a/a) (Köntgen *et al*., 1993) representing 115 different alleles. A total of 6,960 transferred blastocysts produced 943 male chimeras that were set up for breeding for at least 6 weeks and sired 3,518 black pups. We obtained GLT for 144 of 216 clones (66.7%). (The real GLT would be higher if chimeras were bred longer, but as soon as heterozygotes are genotyped for a gene‐targeted ES clone, all chimeras for the same mutation are culled.) In all three configurations, ES cell‐derived offspring are black (+/+ and a/a). In the first configuration, which is our preferred configuration and currently our standard practice, host‐derived offspring would be agouti (*Tyr^c^*/*+* and A/a) but have not been seen among 1,807 offspring (which were instead all black), corroborating the extreme stringency of the cell‐autonomous defect in spermatogenesis of the *Tsc22d3* mutant phenotype. In the second and third configurations, host‐derived offspring would be agouti or black. We identified approximately 50% of heterozygotes among black offspring in the second and third configurations, which is consistent with—but not conclusive of—the absence of leakiness of the *Tsc22d3* mutant phenotype.

**Table 1:** Exclusive Generation of ES Cell‐Derived Offspring with goGermline Technology for Gene‐Targeted Clones from Parental ES Cell Line Bruce4

Donor	ES clones	GLT		Transferred blastocysts	Pups born alive		Chimeras born alive		Chimeras at weaning		Male chimeras		Chimeras mated ≥6 weeks		Fertile chimeras		Offspring
BALB/c × albino‐agouti B6	106	74	(70)	3,708	1,603	(43)	794	(50)	737	(93)	673	(91)	479	(71)	188	(39)	1,807
C57BL/6	95	60	(63)	2,808	1,013	(36)	636	(63)	545	(86)	543	(99)	403	(74)	121	(30)	1,516
BALB/c × C57BL/6	15	10	(67)	444	172	(39)	100	(58)	87	(87)	87	(100)	61	(70)	24	(39)	195
	216	144	(67)	6,960	2,788	(40)	1,530	(55)	1,369	(89)	1,303	(95)	943	(72)	333	(35)	3,518

The percentage in a given column relates to the number in the previous column. Three configurations for blastocyst donors were tested; the efficiency at each step is comparable. Studs were BALB/c males in all three configurations. Female pups were culled soon after birth, explaining why 95% of weaned chimeras are male. Breeding results are listed only for chimeras that were given the opportunity to breed for ≥6 weeks. As soon as GLT was obtained for a particular ES cell clone, all other chimeras for the same mutation were culled in order to keep the total number of mice generated to a minimum. Therefore, if given a longer time period, more than 35% of male chimeras could have produced offspring. This percentage of 35% thus ought to be regarded as a lower estimate. The 216 gene‐targeted ES clones represent 202 different clones; some were used in more than one configuration.

**Figure 3:**
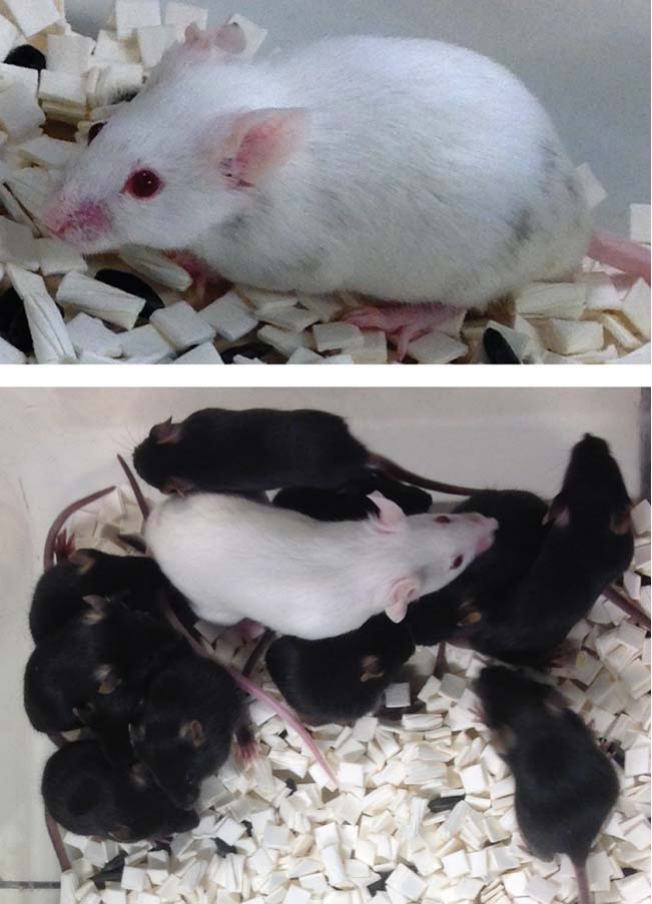
Exclusive transmission of the ES cell‐derived genome through a goGermline chimera with low‐grade coat‐color chimerism. (**Top**) This male chimera was produced by injecting a gene‐targeted ES clone from the Bruce4 parental ES cell line (black) into a goGermline blastocyst (albino). The level of coat‐color chimerism is minor, and would be deemed insufficient for setting up this chimera for breeding if generated with the conventional method. (**Bottom**) This male chimera sired two litters (here pictured together) when bred with C57BL/6 females (not pictured). All offspring are black and are thus ES cell‐derived, demonstrating 100% GLT.

We have found repeatedly that low‐grade coat‐color chimeras, which would not be deemed worthwile setting up for breeding if generated with a conventional method, give 100% GLT (Fig. 3). We surmise that, as there is no competition from host‐derived germ cells, the germline of male goGermline chimeras can be colonized efficiently by germ cells that descended from the injected ES cells, regardless of whether these ES cells resulted in high coat‐color chimerism.

## DISCUSSION

We have here demonstrated that the new goGermline technology can be applied with high efficiency to gene‐targeted clones from the commonly used parental ES cell lines E14, JM8A3.N1, and Bruce4. Fertile male chimeras give exclusive, 100% GLT and behave genetically as heterozygotes. The pseudo‐heterozygous nature of fertile male chimeras is immensely useful for generating mice with a heterozygous phenotype of diminished viability, such as is the case for the *Meis2* knockout. The method does not afford exclusive transmission of the mutation through the male germline. No genotyping of the two constituent strains of the goGermline technology is required. A major advantage over the Perfect Host approach, as it was described (Taft *et al*., 2013), is that the goGermline technology exploits fully the practical advantages of coat color differences for identifying chimeras and ES cell‐derived offspring. Our preferred configuration for blastocyst production is that of homozygous *Tsc22d3* floxed, BALB/c × albino‐agouti C57BL/6 F1 females mated with homozygous *ROSA26‐Cre* BALB/c males. The yield of blastocysts in this hybrid configuration is high and consistent, and the blastocyts are easy to inject. The albino coat color of these hosts lends itself well to assess coat color chimerism visually for ES cell lines derived from genetic backgrounds with a black (Bruce4, JM8), chinchilla (E14), or agouti (JM8A3, W9.5) coat color. Our extensive validation has not revealed any evidence of leakiness, that is, of hemizygous *Tsc22d3* knockout germ cells producing fertile spermatozoa; the sterility is due to a cell‐autonomous defect in spermatogenesis that is extremely stringent. In independent observations, males carrying one of three distinct *Tsc22d3* knockout alleles were reported to be sterile (Bruscoli *et al*., 2012; Ngo et al., 2013b; Romero *et al*., 2012; Suarez *et al*., 2012), consistent with a complete loss of the germline due to an intrinsic failure. Wild‐type germ cells transplanted into the testes of hemizygous *Tsc22d3* knockout males can repopulate seminiferous tubuli (Bruscoli *et al*., 2012), suggesting that these testes can still support normal spermatogenesis. Thus, the testicular environment of hemizygous *Tsc22d3* knockout males and of chimeric males developing from injected goGermline blastocysts is conducive to germ cell differentiation, either from transplanted germ cells (Bruscoli *et al*., 2012) or from germ cells that descended from the injected ES cells (this article). Although there is no reason to doubt the fidelity of sterility, it is cautious to rely on coat color differences when generating chimeras and assessing offspring, if only as quality control for mouse colony management and strain purity.

The complete elimination of the collateral birth of undesired, non‐ES cell‐derived offspring from breeding conventional chimeras fulfills adequately the reduction imperative of the 3R principle of humane experimental technique with animals (Russell and Burch, 1959). In our current standard operating protocol, a project is considered successful as soon as females mated with chimeras are observed to be pregnant; no further injections need to be scheduled, and these or additional chimeras need not be bred further. In preliminary observations, we have found that it is possible to enrich substantially for chimeras that are fertile (and thus give 100% GLT) by orchidometry, thereby avoiding the housing and attempted breeding of chimeras that turn out to be sterile. Because fertile chimeras behave genetically as heterozygotes, they can be bred directly to Cre or Flp‐expressing or other strains, thus eliminating the need for initial colony expansion, hence saving time and further reducing the number of mice used.

## CONCLUSION

Recessive mutations in autosomal male fertility genes could be harnessed to develop similar approaches with 100% GLT, but the breeding scheme would be more complex. The location of *Tsc22d3* on the X chromosome is very convenient.

## METHODS

### Mouse Strains for goGermline Technology

The floxed *Tsc22d3* mutation contains two *loxP* sites that are 1.9 kb apart and flank the last exon of the gene. Gene targeting was carried out in the parental ES cell line Bruce4 (C57BL/6‐Thy1.1 background) (Köntgen *et al*., 1993) with G418 selection; a gene‐targeted ES clone was injected into F2 of BALB/c x albino‐agouti C57BL/6 (*Tyr^c^*/*Tyr^c^* and A/A) blastocysts; and GLT was obtained by crossing chimeras with C57BL/6 mice carrying a *ROSA26‐Flp* mutation in order to excise the *FRT*‐flanked neomycin selectable marker gene. The floxed *Tsc22d3* mutation devoid of the neomycin selectable marker gene was further maintained in a C57BL/6 background; this is the C57BL/6 strain used in the second and third configurations of Table 1. The floxed *Tsc22d3* mutation devoid of the neomycin selectable marker gene was backcrossed four times to BALB/c and then intercrossed to generate a strain that is homozygous for the floxed *Tsc22d3* mutation and that is also homozygous for a knockout *Tyr* allele at the albino locus and the A allele at the agouti locus; this is the BALB/c strain used in the first and third configurations of Table 1. Separately, the floxed *Tsc22d3* mutation devoid of the neomycin selectable marker gene was backcrossed twice to albino‐agouti C57BL/6 mice and then intercrossed to generate a strain that is homozygous for the floxed *Tsc22d3* mutation and that is also homozygous for the *Tyr^c‐2J^* allele at the albino locus and the *A* allele at the agouti locus; this is the albino‐agouti C57BL/6 strain used in the first configuration of Table 1. In albino‐agouti C57BL/6 mice, the wild‐type *Tyr* allele at the albino locus and the nonagouti *a* allele at the agouti locus of C57BL/6 have been replaced with, respectively, the albino allele of B6(Cg)‐*Tyr^c‐2J^*/J (The Jackson Laboratory, stock number 000058), and the agouti *A* allele of BALB/c. The *a* and *A* alleles at the agouti locus were distinguished by Southern blotting from ear biopsy genomic DNA. The *Tyr^c^* and *Tyr^c‐2J^* alleles were not distinguished by genotyping because both are knockout alleles of the tyrosinase gene and are functionally equivalent; they are here referred to as *Tyr^c^*. The *ROSA26‐Cre* mutation was introduced in a BALB/c ES cell line (Noben‐Trauth *et al*., 1996), and established and maintained homozygously in a BALB/c background. Strains are available under license from Ozgene.

### Generation and Breeding of goGermline Chimeras

Homozygous floxed *Tsc22d3* females were superovulated at age 19–25 days with PMSG and hGC administered 47–48 hr apart. At Ozgene, blastocyts were collected at 3.5 days *post coitum*, injected with ES cells, and transferred to pseudopregnant recipients that are an F1 of CBA × C57BL/6. At the Max Planck Research Unit for Neurogenetics, morulae were collected at 2.5 days *post coitum*, and cultured overnight in KSOM medium; blastocysts were injected with ES cells, and transferred to pseudopregnant recipients of strain CD‐1. At Ozgene, most female pups were sacrificed at age 8–10 days, and chimerism of the male pups assessed at 21 days. Males with high coat color chimerism were set up for breeding at age 42 days or later. At Ozgene, these male chimeras were allowed to mate with C57BL/6 females for at least 6 weeks, but when one or more male chimeras gave GLT, the other male chimeras for the same mutation were culled. At Ozgene, all black offspring were genotyped by radioactive Southern blot hybridization. At the Max Planck Research Unit for Neurogenetics, all offspring and embryos were genotyped, by non‐radioactive Southern blot hybridization or the polymerase chain reaction.

### Gene Targeting at the *Omp* Locus and at the *Meis2* Locus

To create the bicistronic OMP‐IRES‐tauGFP targeted mutation, the *Omp* coding sequence was generated by DNA synthesis (GeneArt), and an *Asc*I site was inserted three nucleotides after the stop codon. This synthetic DNA fragment was inserted into generic *Omp* targeting vector pPM9 (Mombaerts *et al*., 1996), which lacks the *Omp* coding sequence. The *IRES‐tauGFP‐ACNF* cassette was inserted into the *Asc*I site. Gene targeting was carried out in the parental ES cell line E14 (129P2/OlaHsd background), and 90 out of 144 G418‐resistant clones (63%) were found to have undergone homologous recombination by genomic Southern blot hybridization. The *ACNF* cassette (Bozza *et al*., 2002; Bunting *et al*., 1999) contains the neomycin selectable marker gene that is self‐excised by the Cre/loxP system in the male germline. The MGI allele name is OMP^tm17Mom^. ES cell clone EPD0413_2_B07 was generated by the trans‐NIH Knockout Mouse Project (KOMP) (Bradley *et al*., 2012) as IKMC project 26474 with targeting vector DPGS00176_A_D03, and obtained from the KOMP Repository at UC Davis. This ES cell clone was derived from the parental ES cell line JM8A3.N1, and is of the type reporter‐tagged deletion allele (with selection cassette) without conditional potential. The MGI allele name is Meis2^tm1(KOMP)Wtsi^. Strains will be available to the research community.

For non‐radioactive Southern blot analysis, liver genomic DNA and clone DNA were extracted with Promega Wizard genomic DNA purification kit, and digested overnight with *ApaL*I. The 500 basepair probe was synthesized with the PCR DIG Probe Synthesis kit (Roche) using the primers 5′*GGAGTCCTGCTATCCTGGA*3′ and 5′*GCTCTGGCCACAGCAACTCA*3′. Probe hybridization was overnight in DIG Easy Hyb solution (Roche). The CDP‐Star alkaline phosphatase substrate (Roche) was used for probe detection according to the manufacturer's guidelines.

### Ethics Statement

All animal studies were carried out in compliance with ethical regulations in Australia and Germany. At Ozgene, mouse experiments were carried out in compliance with the Australian Code of Practice for the Care and Use of Animals for Scientific Purposes. Approval came from the Ozgene Animal Ethics Committee. At the Max Planck Research Unit for Neurogenetics, mouse experiments were performed in accordance with the German Animal Welfare Act, the European Communities Council Directive 2010/63/EU, and the institutional ethical and animal welfare guidelines of the Max Planck Research Unit for Neurogenetics. Approval came from the *Regierungspräsidium* Darmstadt and the *Veterinäramt* of the City of Frankfurt.

## AUTHOR CONTRIBUTIONS

F.K. designed the research and analyzed the data. J.L., M. Katidou, I. Chang, and M. Khan performed experiments. J.W. coordinated research and analyzed the data. P.M. supervised the research in his laboratory, managed the collaboration with Ozgene, and wrote the manuscript. All of the authors contributed to editing the manuscript.
